# The in vitro research of bacterial invasion of prosthetic vascular grafts: comparison of elastomer-sealed and gelatin-coated Dacron vascular grafts

**DOI:** 10.1007/s00595-013-0761-8

**Published:** 2013-10-20

**Authors:** Yuki Sasaki

**Affiliations:** Division of Cardiovascular Surgery, Department of Surgery, School of Medicine, Faculty of Medicine, Toho University, 6-11-1 Omori-nishi, Ota-ku, Tokyo, 143-8541 Japan

**Keywords:** Graft infection, Bacterial invasion, Elastomer-sealed Dacron vascular graft, Gelatin-coated Dacron vascular graft, *Pseudomonas aeruginosa*

## Abstract

**Purpose:**

To investigate the process of bacterial invasion from the surface to inside prosthetic vascular grafts.

**Methods:**

Elastomer-sealed Dacron vascular grafts (ESDVGs) and gelatin-coated Dacron vascular grafts (GCDVGs) were cut into 6-cm segments and placed in a U-shaped configuration on culture plates. Physiological saline was poured inside the grafts and a suspension of *Pseudomonas aeruginosa* was added to the outside. Samples taken from inside the grafts at nine time points for up to 60 h were spread on agar. Bacterial colonies were then analyzed. The grafts were also examined using scanning electron microscopy (SEM).

**Results:**

Contaminated vascular graft models were produced in 18 ESDVGs (group T) and 12 GCDVGs (group G). The bacterial counts inside the vascular grafts in both groups increased over time. Bacterial colonies were confirmed in all samples from group G by 30 h, whereas bacteria appeared inside the grafts from group T at various times between 0 and 60 h. Bacteria were undetectable in one model from group T throughout the study. SEM revealed that the elastomeric membrane in the ESDVG was uneven.

**Conclusion:**

Bacterial invasion of vascular grafts does not always occur immediately after contamination. ESDVGs may be more resistant to bacterial invasion as they have a thicker and evenly enriched elastomeric membrane.

## Introduction

Infection of a prosthetic vascular graft is a serious complication, which can progress to lethal systemic sepsis if treatment is delayed. Thus, the factors involved in the infectivity of prosthetic vascular grafts should be established in relation to bacterial adherence [1] and invasion. Regarding bacterial adherence to vascular grafts [1] and antibiotic-coated grafts [2, 3], some in vitro and in vivo [4] studies have been published. We investigated bacterial invasion from the outer surface to the inside of vascular grafts using a new experimental system in vitro to consider the relationships between the time required for invasion and the amount of bacteria inside the grafts. Graft fragments were also examined using scanning electron microscopy (SEM). We compared classical gelatin-coated Dacron vascular grafts (GCDVGs) with new elastomer-sealed Dacron vascular grafts (ESDVGs) to elucidate the effects of bacterial invasion on different materials. Increased knowledge about graft infectivity will assist in the treatment of this refractory complication and help avoid implanted graft excision.

## Methods

The Committee for Biosafety and Biosecurity at Toho University School of Medicine (Tokyo, Japan) approved the study protocol. Experiments using virulent bacteria were carried out in a laboratory at the Department of Microbiology and Infectious Diseases (School of Medicine, Faculty of Medicine, Toho University, Tokyo, Japan) under the guidance of qualified staff.

### Prosthetic vascular grafts

Straight, 8-mm diameter ESDVGs (Triplex^®^, Vascutek Terumo, Tokyo, Japan) and GCDVGs (Gelweave^®^, Vascutek Terumo, Tokyo, Japan) were cut into 6-cm segments.

### Model bacterial strain and suspension

The model bacterium was *Pseudomonas aeruginosa* strain PAO1 (ATCC 15692) because of its motility and ability to thrive in physiological saline. *P. aeruginosa* frozen at −80 °C was spread on LB agar and incubated for 18 h at 35 °C. Resulting bacterial colonies were transferred, using a sterile loop, into 30 mL of LB broth and incubated for 24 h at 35 °C. Thereafter, 3 mL of the LB broth containing *P. aeruginosa* was centrifuged at 8,000 rpm for 5 min and the sediment was resuspended in 30 mL of physiological saline and used as the bacterial suspension.

The bacterial concentration in the suspensions before the experiments was confirmed to be approximately 1.0 × 10^8^ colony forming units (CFU)/mL by spreading 10 μL of each properly diluted bacterial suspension on LB agar and counting the colonies manually after 18 h of incubation at 35 °C. Bacterial suspensions were maintained by shaking at 160 rpm at 35 °C until use.

### Study 1: Relationship between time required for invasion and the density of bacteria inside the vascular grafts

#### Models of contaminated vascular grafts

**Fig. 1:**
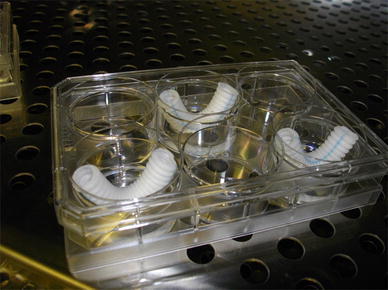
Establishment of contaminated vascular graft models

The 18 ESDVG (group T) and 12 GCDVG (group G) models of contaminated vascular grafts were produced as follows (Fig. 1): Prosthetic vascular grafts cut into 6-cm segments were placed in a U-shaped configuration in six-well culture plates (FALCON^®^, Becton, Dickinson and Co., Franklin Lakes, NJ, USA). Sterile physiological saline (2 mL) was poured inside the grafts and bacterial suspensions (5 mL) were added to the area outside the grafts. The plates were covered and incubated at 35 °C until the end of the study.

#### Sampling and bacterial counting

Samples (50 μL) collected from the inside of the grafts using micropipettes were appropriately diluted, spread on LB agar, and incubated at 35 °C for 18 h. Bacterial colonies were then manually counted. Samples were collected every 6 h for 36 h, and then 48 and 60 h from inside, and 0, 24, 48 and 60 h from outside the grafts. Bacterial counts are expressed as CFU/mL and were converted into log CFU/mL for statistical analysis. The limit of bacterial detection was 2.0 × 10^1^ CFU/mL.

### Statistical analysis

Arithmetic data are expressed as mean ± standard deviation and were statistically analyzed using JMP 9.0.3 (SAS Institute Inc., Cary, NC, USA). Bacterial counts inside and outside the vascular grafts at each time point between two groups were evaluated using the Mann–Whitney *U* test. The time elapsed before bacteria appeared inside the vascular grafts was calculated from Kaplan–Meier curves and then values were compared between the groups using the log-rank test. A *P* value < 0.05 was considered to indicate a significant difference.

### Study 2: Observations of prosthetic vascular grafts by SEM

An S-3500N Scanning Electron Microscope (Hitachi Ltd., Tokyo, Japan) was used to examine the structures of fresh ESDVGs and GCDVGs and determine the degrees of invasion of the graft walls at various angles after incubation for 60 h during the study 1 in suspensions of *P. aeruginosa*.

#### Sample processing for SEM

Vascular grafts were fixed in 2.0 % glutaraldehyde for 48 h, rinsed in physiological saline, fixed with 2.0 % osmium tetroxide for 2 h, irrigated with distilled water and then dehydrated in a graded series of 50–100 % ethanol. They were then placed in tertiary-butyl alcohol and dried in an ES-2030 critical-point dryer (Hitachi Ltd., Tokyo, Japan). Dried grafts were trimmed, mounted onto SEM slabs at various angles and sputter-coated with platinum-vanadium for 80 s using an Ion Sputter E-1030 (Hitachi Ltd., Tokyo, Japan). Graft structures and degrees of bacterial invasion were investigated as described above in long-axis and short-axis views.

## Results

### Study 1

Bacteria started to invade the GCDVG from 6 h post-immersion and all samples from inside the GCDVGs had generated bacterial colonies by 30 h. The average elapsed time until bacteria appeared inside the GCDVGs was 15.5 ± 7.0 h. Bacteria were identified inside two ESDVGs immediately after immersion, and in one model after 60 h. Bacteria remained undetectable in another model throughout the study. The average time taken for bacteria to become detectable inside the ESDVGs was 22.0 ± 19.7 h.

**Fig. 2:**
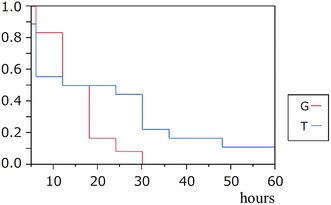
*Vertical axis* shows ratio of models in which bacteria were undetectable inside vascular grafts. The *horizontal axis* shows elapsed time. Bacteria were detected inside grafts in all models of group G (GCDVG group) by 30 h. Bacteria were undetectable throughout the study in one model from group T (ESDVG group). However, log-rank test did not reveal any significant differences between the two groups

Bacterial counts inside the grafts increased over time in both groups. Bacterial counts outside (Table 1) and inside (Table 2) the vascular grafts at each time point did not differ significantly between the groups (*P* > 0.05, Mann–Whitney *U* test). The time that elapsed before bacteria appeared inside the vascular grafts was determined from Kaplan–Meier curves (Fig. 2). Values did not differ significantly between the groups (*P* > 0.05, log-rank test).

**Table 1:** Average bacterial counts outside the vascular grafts

Bacterial counts (log CFU/mL)			
Elapsed time (h)^a^	Group G	Group T	
0	7.0 ± 0.4	7.2 ± 0.6	NS
24	7.5 ± 0.3	7.3 ± 0.4	NS
48	7.5 ± 0.3	7.6 ± 0.3	NS
60	7.7 ± 0.2	7.7 ± 0.4	NS

Bacterial counts are expressed as mean ± standard deviation

Mann–Whitney *U* test of data at each time point revealed no significant differences (NS) between group T (ESDVG group) and group G (GCDVG group)

^a^Hours after establishing models of contaminated vascular grafts

**Table 2:** Average bacterial counts inside the vascular grafts

Bacterial counts (log CFU/mL)			
Elapsed time (h)^a^	Group G	Group T	
0	0	0.1 ± 0.4	NS
6	0.3 ± 0.6	1.2 ± 1.5	NS
12	1.0 ± 1.1	1.2 ± 1.7	NS
18	2.6 ± 1.5	1.8 ± 2.0	NS
24	3.6 ± 1.4	2.4 ± 2.3	NS
30	4.4 ± 0.9	3.4 ± 2.3	NS
36	4.6 ± 1.1	3.8 ± 2.4	NS
48	4.9 ± 1.0	4.1 ± 2.5	NS
60	5.1 ± 1.0	4.5 ± 2.5	NS

Bacterial counts are expressed as mean ± standard deviation

Mann–Whitney *U* test of data at each time point revealed no significant differences (NS) between group T (ESDVG group) and group G (GCDVG group)

^a^Hours after establishing models of contaminated vascular grafts

### Study 2

**Fig. 3:**
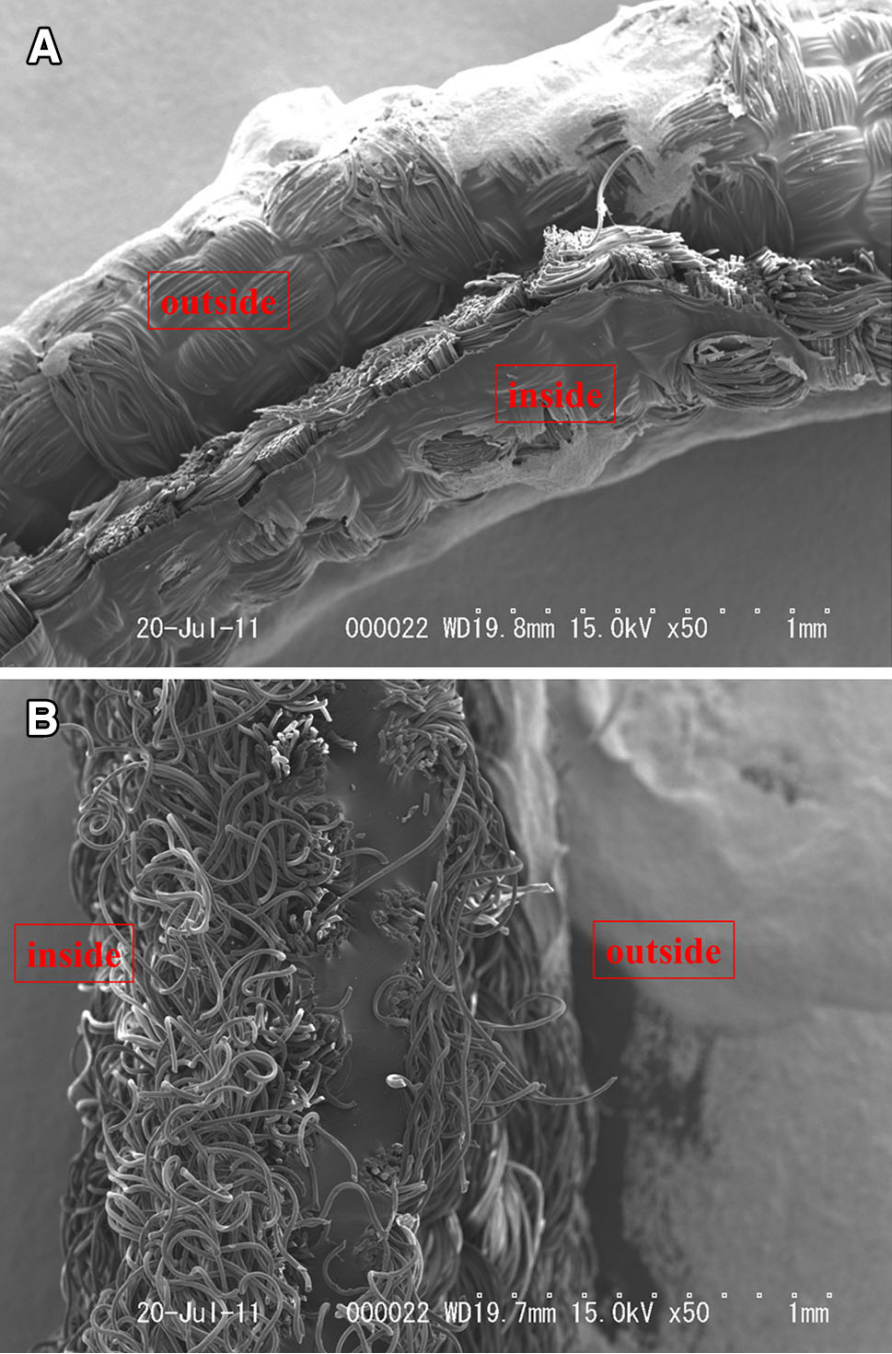
**a** Scanning electron microscopy finding of sterile gelatin-coated Dacron vascular graft. Short-axis view of gelatin-coated Dacron vascular graft shows woven Dacron structure (magnification ×50). **b** Scanning electron microscopy finding of sterile elastomer-sealed Dacron vascular graft. Short-axis view of elastomer-sealed Dacron vascular graft shows unique three-layer structure comprising a central elastomeric membrane sandwiched between layers of knitted Dacron (magnification ×50)

**Fig. 4:**
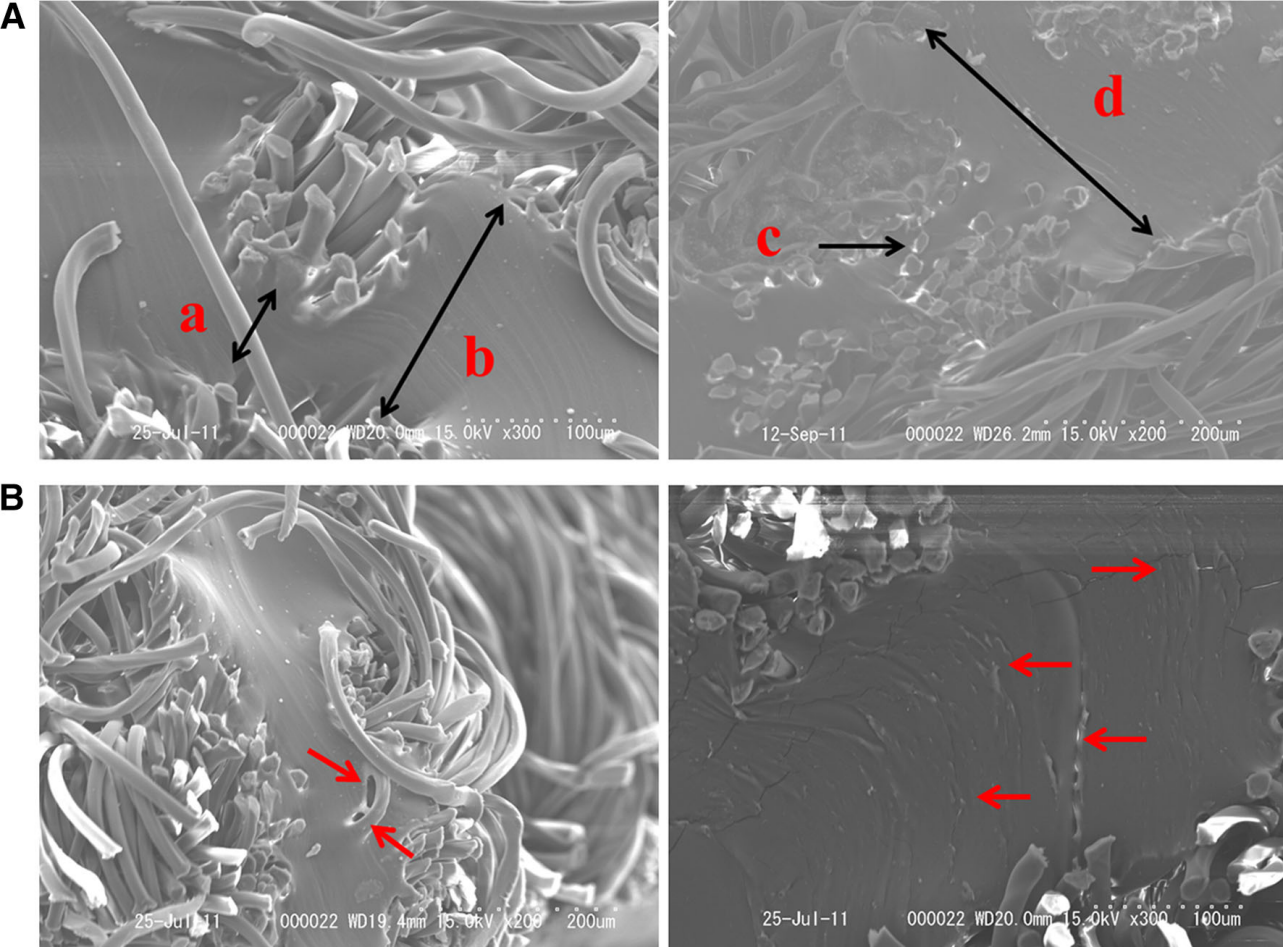
**a** Scanning electron microscopy findings of sterile elastomer-sealed Dacron vascular grafts. Short-axis views at ×300 (*left*) and ×200 (*right*) magnification show uneven thickness of elastomeric membrane. *Arrows*: *a*, 50 μm; *b*, 150 μm; *c*, 2 μm; *d*, 300 μm. **b** Scanning electron microscopy findings of sterile elastomer-sealed Dacron vascular grafts. Short-(*left*) and long-(*right*) axis views at magnification of ×200 and ×300, respectively, show defects in elastomeric membrane (*arrows*)

Figure 3a, b shows short-axis SEM views of a sterile GCDVG and ESDVG, respectively. The wall of the ESDVG comprised a central, low-porosity elastomeric membrane sandwiched between outer and inner layers of knitted Dacron. The elastomeric membrane was proved to have uneven thickness and the thinnest portion was about 2 μm (Fig. 4a). Defects were also evident in the membrane (Fig. 4b). The uneven thickness and defects in the elastomeric membrane might have been associated with the time required for the bacteria to invade the ESDVG.

**Fig. 5:**
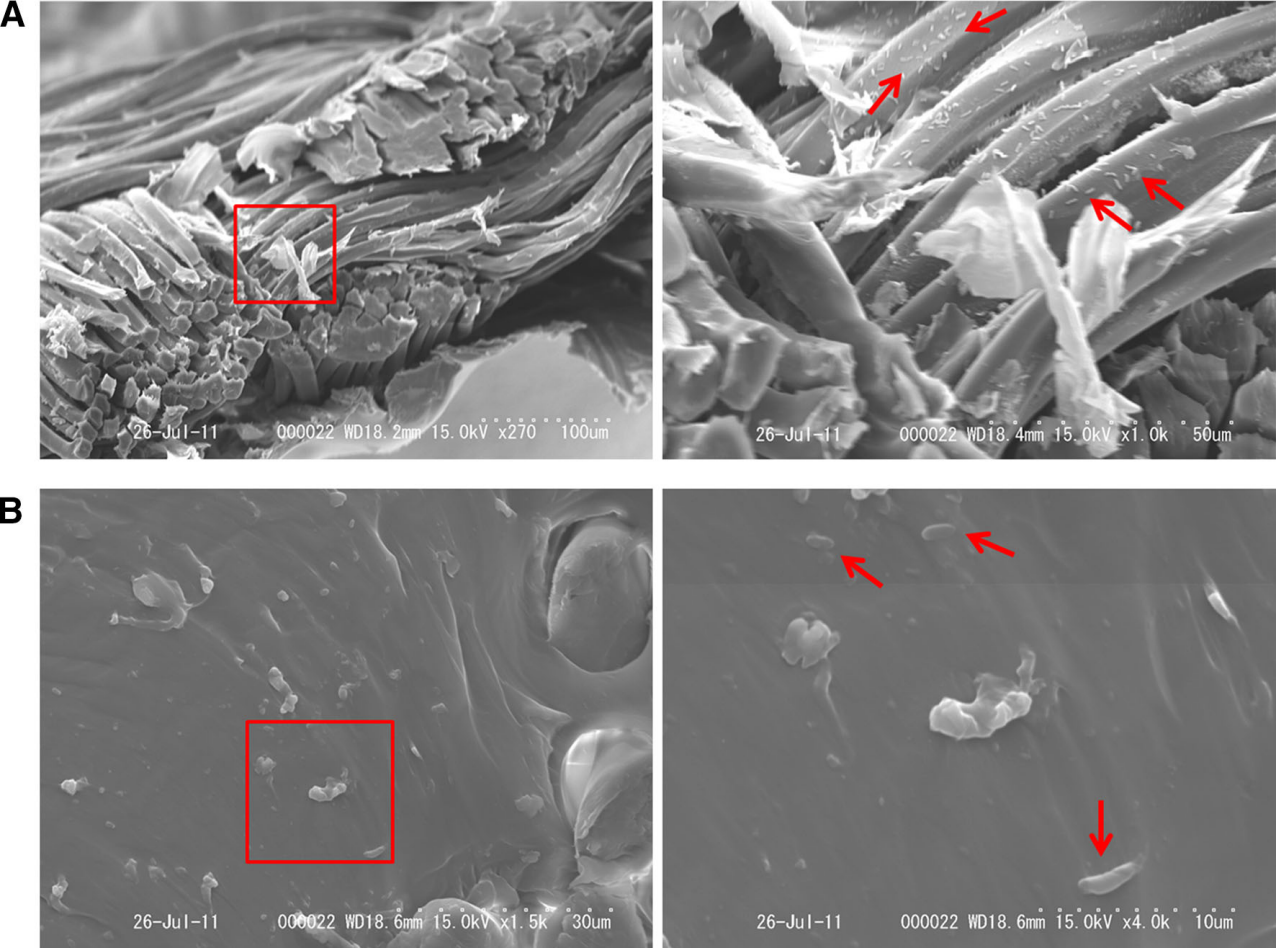
**a** Scanning electron microscopy views of gelatin-coated Dacron vascular graft after immersion in *Pseudomonas aeruginosa* suspension for 60 h. There are long-axis views of the fragment of gelatin-coated Dacron vascular graft at magnification of ×270 (*left*) and ×1000 (*right*). Many bacteria are evident in gaps between graft fibers at ×1000 magnification. **b** Scanning electron microscopy views of elastomer-sealed Dacron vascular graft after immersion in *Pseudomonas aeruginosa* suspension for 60 h. There are short-axis views of elastomeric membrane in fragment of elastomer-sealed Dacron vascular graft at magnification of ×1500 (*left*) and ×4000 (*right*). Bacteria are evident in elastomeric membrane (*arrows*)

SEM views of grafts inoculated with *P. aeruginosa* for 60 h revealed bacteria in gaps between the GCDVG fibers (Fig. 5a) and in the elastomeric membrane of the ESDVGs (Fig. 5b).

## Discussion

The model bacterial species in this study was *P. aeruginosa* because of its motility and ability to exist in physiological saline. Although it is not a typical bacterium found in vascular graft infection, there are reports of aortic aneurysm and prosthetic vascular graft infections caused by *P. aeruginosa* [5–7]. Furthermore, the indications for major aortic and peripheral vascular surgery have recently been extended to include high-risk patients, making the incidence of infection caused by *P. aeruginosa* likely to increase. In fact, it has already become a serious issue, especially during the vascular surgery [8]. Thus, vascular graft infection caused by this organism should be investigated.

The new ESDVG used in the present study has a unique structure comprising a central elastomeric membrane sandwiched between two layers of high-porosity knitted Dacron [9, 10]. The elastomeric membrane consists of a highly flexible styrene polymer with a low-porosity material. The porosity of the ESDVG is significantly lower than that of standard coated grafts, preventing water leakage [9]. Accordingly, I postulated that the ESDVG would be more resistant to bacterial invasion than standard coated vascular grafts when exposed to direct external bacterial contamination. I selected gelatin-coated woven Dacron vascular grafts to compare with the ESDVGs. In the clinical setting, gelatin-coated woven Dacron grafts are used more frequently than knitted Dacron grafts due to enlargement of the diameter during the postoperative course [11]. Coated knitted Dacron vascular grafts are not used in our institution even for surgery on the abdominal aorta. The present study investigated only vascular grafts that are applied in clinical medicine.

Although no significant differences were identified between the two types of grafts, the time that elapsed before bacteria appeared inside the ESDVGs ranged from 0 to 60 h and SEM confirmed thin areas and defects in the elastomeric membrane of the sterile ESDVGs. These features might have been associated with the immediate appearance of bacteria inside the ESDVG. The thinner areas and defects in the elastomeric membrane may have facilitated bacterial invasion. If so, this could be prevented by thickening and enriching the elastomeric membrane.

No bacterium was confirmed in samples from inside the GCDVGs at 0 h. According to experimental data presented by Terumo Corporation (Tokyo, Japan), which simulates 14 days of in vivo hydrolysis, the gelatin remaining rates on the grafts are about 100, 90, 80, 70, and 5 % on days 0, 2, 4, 7, and 14, respectively. Although my experiment is in vitro, basic research, time and the amount of bacterial invasion into vascular graft would be minimally affected by the hydrolysis rate of the gelatin sealant. The present in vitro results also indicate that bacterial invasion into vascular grafts is not always immediate, even if the grafts are located in a highly contaminated area. As bacterial counts inside vascular grafts would increase over time, prosthetic vascular grafts implanted in infected wounds should be drained and irrigated as soon as possible to prevent systemic sepsis and graft removal in the clinical setting. Recent clinical efforts have been made to preserve infected prosthetic vascular grafts. Dosluoglu and colleagues [12] argue that patients with exposed grafts after inguinal wound dehiscence without systemic sepsis can be treated by graft preservation treatment such as negative pressure wound therapy (NPWT). Under probable contaminated conditions such as traumatic disease or infected aneurysms, vascular grafts should be made resistant to bacterial invasion by sufficient irrigation and the insertion of a suction drain around them. NPWT at wound dehiscence would also help prevent graft infection and systemic sepsis.

In conclusion, the findings of the present study suggest that evenly thickening and enriching the elastomeric membrane of ESDVGs may improve their ability to resist or suppress bacterial invasion. To my knowledge, this is the first published investigation of the mechanism of bacterial invasion into prosthetic vascular grafts.
